# Breeding of African sheep reared under low-input/output smallholder production systems for trypanotolerance

**DOI:** 10.14202/vetworld.2022.1031-1043

**Published:** 2022-04-23

**Authors:** Dikeledi P. Malatji

**Affiliations:** Department of Agriculture and Animal Health, College of Agriculture and Environmental Sciences, University of South Africa, Johannesburg, Gauteng Province, South Africa

**Keywords:** Trypanotolerance, animal breeding, small ruminants, trypanocidal drugs

## Abstract

Trypanosomiasis is a disease caused by unicellular protozoan parasites. Small ruminants succumb to trypanosomiasis in areas of high tsetse fly challenge, resulting in serious economic loss often to farmers in low-input smallholder systems. At present, trypanosomiasis is treated with trypanocidal drugs, but access to these can be limited, and increasing parasite resistance raises questions about their efficacy. The development of trypanotolerance in small ruminant flocks through targeted breeding strategies is considered a sustainable and economical option for controlling African trypanosomiasis. Recently, quantitative trait loci (QTLs) associated with trypanotolerance traits in sheep have been reported. The results of these studies form the basis for more studies to identify QTLs associated with trypanosomiasis resistance, particularly in African livestock species. For example, signatures of positive selection for trypanotolerance have been identified using genome-wide single-nucleotide polymorphism data. However, there are several challenges in performing genetic analyses using data from low-input smallholder systems, including a lack of recorded pedigree and production records and the need for large sample sizes when flock sizes are often fewer than 50 animals. Breeding strategies to improve trypanotolerance should also preserve existing genetic diversity as well as minimize excessive genetic introgression by trypanosusceptible breeds. This review discusses the possibilities of breeding for trypanosome tolerance/resistance in low-input/low-output small ruminant production systems. Potential challenges are outlined, and potential available genetic resources are described as a foundation for future work.

## Introduction

**Figure-1:**
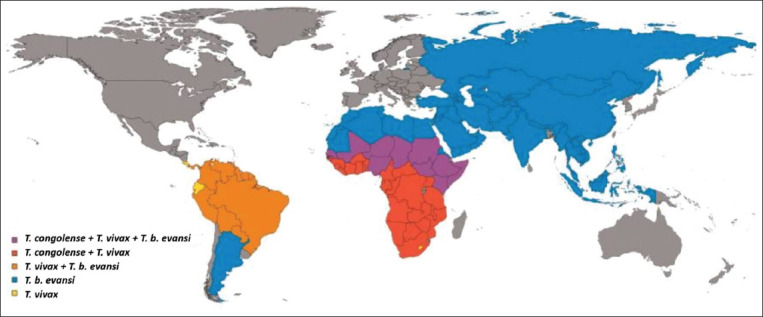
Map showing countries where livestock trypanosomes of importance are recorded [6].

Trypanosomiasis is a disease caused by unicellular protozoan parasites of the order Kinetoplastida, family Trypanosomatidae, genus *Trypanosoma*. Trypanosomiasis affects the health of humans [1] and livestock to varying degrees. In sub-Saharan Africa, trypanosomiasis is estimated to cause yearly losses of over 4.5 billion US dollars through indirect and direct production costs [2,3]. The disease is widespread in sub-Saharan Africa (Figure-1) and is referred to as African Animal Trypanosomiasis (AAT) [3,4] transmitted by the tsetse fly (*Glossina* spp.) [5,6].

AAT, also called nagana, is the major constraint on livestock production in African countries. It is caused mainly by *Trypanosoma vivax*, *T. congolense*, and *Trypanosoma brucei brucei* [7,8]. The majority of sheep in rural areas of Africa are raised under low-input/low-output production systems [9]. In most areas, this production system is characterized by poor-quality feeds, shortage of drinking water, poor husbandry, poor management, and scarcity of feed resources, resulting in substandard sheep management practices by small-scale farmers [10]. A study by Aliber and Hall [11] attributed the obstacles to improved sheep productivity to poor interactions among farmers, extension workers, researchers, and policy makers. Nonetheless, sheep raised in these production systems are prone to diseases and parasites [12,13].

The challenge of AAT varies with geographic location and seasonal changes [14]. Temperature, as one of the aspects of climate, has been shown to influence the growth and proliferation of trypanosomes within the tsetse fly [15]. The strong correlation between maximum temperature and trypanosome prevalence was confirmed by Nnko *et al*. [14]. Animals infected with trypanosomiasis show symptoms such as chronic anemia [16], neurological symptoms, enlarged lymph nodes [6], reduced productivity, abortion, impaired fertility [17,18], weakness, and emaciation [19]. Trypanosomes can cause immunosuppression with concurrent infections complicating AAT. If the disease is not treated, it can ultimately lead to death [6]. Animals that have recovered clinically may relapse when stressed.

Trypanosomiasis causes significant financial losses associated with controlling the effects of AAT. In Africa, communal farmers in rural areas resort to using trypanocides to protect their livestock due to lack of governmental intervention to introduce and sustain African trypanosomiasis control programs [20]. Treatment of infected animals can be costly for the farmer because AAT is known to be a herd health problem. Further, some farmers are resource-constrained and do not have access to drugs used to treat livestock for trypanosomiasis. The cost of treatment under low tsetse challenge may be significantly less than that of the prophylactic treatment of the entire herd [21]. Targeted breeding strategies offer a viable and more sustainable option compared with the use of trypanocides.

This review summarizes the available information on trypanosome tolerance/resistance in low-input/low-output production systems in Africa.

## Effects of Trypanosomiasis in Small Ruminants

Not many studies have been performed in Africa on the effect of trypanosomiasis in small ruminants. A study from several decades ago reported that small ruminants survive much better under medium tsetse challenges than do cattle [22]. Later studies have shown that small ruminants succumb to trypanosomiasis in areas of high tsetse challenge, resulting in serious economic losses [23,24]. According to Masiga *et al*. [23], trypanosomiasis causes anemia in small ruminants in Kenya. In addition, trypanosome infections lead to heme catabolism and erythrophagocytosis, resulting in iron accumulation in the tissues, hyperbilirubinemia, liver dysfunction, and multiple organ failure [25]. Hemorrhage, pulmonary edema, atrophy of body fat, and pulmonary edema are observed on postmortem examination of animals that died from trypanosome infection. A study by Karanja [26] reported that livestock with AAT were likely to die (mean case fatality rate, 67-90%). These studies provide a crucial basis for studying the significance of trypanosomiasis in small ruminants.

## Detection of Trypanosomiasis in Small Ruminants

Researchers have used microscopic techniques to diagnose trypanosomiasis in small ruminants to detect trypanosome infection with limited sensitivity [27]. Microscopic diagnosis is laborious and requires a large number of trypanosomes for accurate detection. Moreover, microscopic techniques fail to detect mixed infections, do not identify trypanosomes that are only found in the midgut, and do not allow the identification of trypanosome species below the subgenus level [28]. However, studies using microscopic techniques to detect trypanosomiasis in animals have established a crucial basis for investigations into the significance of this disease in small ruminants, particularly sheep.

In recent years, molecular biological methods have overcome these limitations, particularly the limits of sensitivity and specificity experienced when using microscopic examination. The introduction of polymerase chain reaction (PCR) brought about a great change in the field and allowed the amplification of specific DNA sequences *in vitro* [29]. Before the introduction of this method, DNA probing and PCR associated with DNA probing methods were used. This method facilitates the detection of trypanosomes [30] and many other types of parasites [31,32]. PCR was evaluated for its ability to detect *T. vivax* DNA in experimentally infected animal blood as early as 5 days post-infection [33]. This study showed that PCR was able to reveal infection in 75% of blood samples. Furthermore, the results showed that PCR amplification of the genomic DNA of *T. vivax* was superior to antigen detecting enzyme-linked immunosorbent immunoassay for the detection of *T. vivax*. In experimental infections, the detection of trypanosome species on buffy coat samples of sheep experimentally infected with *T. vivax*, sampled daily, had an overall sensitivity of 13% by microscopic observation, compared with 40% by PCR [33]. However, because of the diversity of *Trypanosoma* species potentially present in a single host, performing PCR diagnosis on host material requires several PCR reactions. To circumvent these limitations, studies have been performed based on the amplification of the internal transcribed spacer-1 (ITS-1) of ribosomal DNA [34,35]. The ITS-1 has the advantage of being a multicopy locus (100–200) in addition to having a small size (300-800 bp), which differs from one taxon to another. This led to the development of a multi-species-specific diagnostic protocol using a single PCR and increased the number of *Trypanosoma* species that could be detected [34]. Moreover, single PCR is used to identify almost all trypanosome species [36]. A detailed review of the applications of PCR-based tools for the detection and identification of animal trypanosomes was given by Desquesnes and Davila [37]. The review showed that molecular tools have many benefits over the use of microscopy alone and can facilitate the identification of different trypanosome species and provide information at scale with minimal labor for epidemiological studies [38].

## Control Methods for Trypanosomiasis

Control methods, such as the use of trypanocidal drugs, insect traps, and insecticides and reducing the proximity of livestock to reservoir hosts [6], have been applied to control trypanosomiasis for several decades. These controlling interventions have not been successful in eradicating the disease. In addition, inappropriate drug usage has resulted in resistance to trypanocidal drugs. Seventeen sub-Saharan African countries reported resistance to veterinary trypanocide drugs in 2008, and the number of countries increased to 21 by early 2015 [39]. It has always been believed that resistance to drugs used in the treatment of trypanosomiasis is caused by gene mutations in the TbAT1/P2 transporter and the high-affinity pentamidine transporter genes responsible for diminazene transport [40]. Studies have shown that treating livestock with trypanocides is dependent on the risk of AAT infection and seasonal variation in tsetse populations. Therefore, AAT control programs need to focus on seasonal differences when tsetse challenge is highest [41].

The cost of new drug discovery has resulted in many pharmaceutical companies losing interest and being reluctant to invest in this approach [42]. Breeding strategies between trypanosusceptible and trypanotolerant animals have been suggested as a means to control the spread of trypanosomiasis [43]. Trypanotolerance is defined as a reduction of the deleterious effects of pathogen burden on the host [44]. Trypanoresistance is defined as a mechanism mediated by the immune system aimed at reducing pathogen burden following infection [44], whereas trypanosusceptibility is defined as the likelihood that an animal will die as a result of trypanosomiasis. Hanotte *et al*. [45] crossbred trypanosusceptible Boran and trypanotolerant N’Dama breed to produce an F2 population that shows heterosis. They found that an F2 cross between susceptible and tolerant breeds had the potential to produce a trypanotolerant synthetic breed that could perform better than either parent under trypanosome challenge. This approach could be a promising strategy to control trypanosomiasis in low-input/low-output small ruminant production systems. To achieve this, a greater understanding of the level of resistance to trypanosomiasis in different breeds of African small ruminants will be required.

## Trypanotolerance in Livestock

Tolerance is a mechanism for defending hosts against pathogens and parasites [46]. Trypanotolerance is defined as the ability of an animal to manage the proliferation of the *Trypanosoma* parasites and prevent their effect on the hosts [47]. Murray *et al*. [48] defined trypanotolerance as the ability of some livestock breeds to survive, reproduce, and produce meat and milk in trypanosome-infested areas where other breeds are unable, without recourse to the use of chemical drugs. In AAT, the criteria used to define trypanotolerance are parasitemia, packed cell volume (an indicator of anemia), and body weight [49]. Trypanotolerance results from different biological mechanisms under multigenic control that is related either to control of the pathogenic effects of the parasite, with anemia considered the most important immunopathological disease-related factor and the major cause of death from trypanosomiasis, or to control of trypanosome infection, as measured by parasitemia [16,50]. Trypanotolerant animals limit anemia through an innate mechanism that controls growth and the hematopoietic system [51].

Unlike cattle, little research has been conducted on small ruminants to identify the mechanisms of trypanotolerance. In cattle, trypanotolerance is defined as the ability of an animal to control the development of severe anemia, which is presumed to be independent of parasitemia levels [16,52]. Using chimeric studies between trypanotolerant N’Dama and trypanosusceptible Boran, Naessens *et al*. [53] showed that trypanotolerance is composed of two partly independent mechanisms, the ability of the hemopoietic system to control anemia [54] and a natural ability to control parasitemia, which is independent of the genetic origin of the hematopoietic tissue. There are indications that these mechanisms may be present in Djallonke sheep [55]; however, more studies are needed on this subject in different sheep breeds. Although there is evidence that host genetic factors play a crucial role in determining an individual’s resistance to trypanosome infection, it should be noted that other factors, such as the species of *Trypanosoma*, nutrition, and the age of the host, can contribute to trypanotolerance [56-59].

Trypanotolerance has been described as a genetically determined complex mechanism [51]. Studies in mice and other animals have utilized molecular techniques to identify genomic regions responsible for controlling trypanotolerance [45,60]. A study in mice identified genes found within quantitative trait loci (QTLs), leading the way to better identify and understand genes controlling trypanotolerance [61,62]. A study by Hanotte *et al*. [45] found 10 trypanotolerant QTLs on mouse chromosomes from a genome-wide scan based on 477 markers. The authors suggested that when selecting Boran and N’Dama cattle for trypanotolerance within an F2 cross, they were able to produce a breed with higher levels of trypanotolerance than the existing parental breeds [45]. A study by Koudande *et al*. [63] used marker-associated introgression to transfer trypanotolerance QTLs from a donor mouse strain into a recipient mouse strain. The study showed that the introgressed mice had improved survival time to challenge than the recipient mice. There is a need to identify the complete pool of genes involved in trypanotolerance in small stock to help design field selection studies by introgression programs.

Most data on the immune response in trypanotolerant animals are derived from experiments using N’Dama cattle. The data show that there is no difference between trypanotolerant and trypanosusceptible cattle in the antibody response to the variable surface glycoproteins of trypanosomes [51]. However, other studies have found significant differences in antibody titers to non-surface-exposed antigens [64] that may be involved in the neutralization of pathogenic molecules of parasite origin, thus contributing to trypanotolerance. There is a dearth of information from similar studies in small ruminants; however, Goossens *et al*. [55] revealed that Djallonke sheep had significantly higher titers of anti-trypanosomal antibody than did Djallonke–Sahelian crossbreeds.

## Breeding livestock for Trypanotolerance/Trypanoresistance

Selective breeding of small ruminants for trypanotolerance could provide a partial solution for low-input/low-output small ruminant production systems in tsetse-infested areas. Breeding livestock for disease resistance [65] is a mainstay of livestock production around the globe, and this subject has been extensively studied in breeds such as the Scottish Blackface Sheep [66]. Breeding programs for disease resistance in sheep have been initiated in countries such as New Zealand and Australia [67]. Studies of livestock production systems in Africa have shown that trypanotolerant breeds [66] are often locally adapted breeds. Measures need to be put in place to preserve genetic diversity when breeding for disease resistance and to protect trypanotolerant local breeds from excessive genetic introgression by trypanosusceptible breeds that may be introduced into a system for production purposes [68]. Some of the evidence for genetic resistance to trypanosomiasis in small ruminants was highlighted by Toure [69]. This study showed that indigenous West African Dwarf goats and Djallonke sheep were trypanotolerant, a finding supported by experimental evidence in sheep. Similarly, there is evidence that indigenous breeds of goats and sheep in East Africa are more resistant to trypanosomiasis than are exotic breeds [70]. Experimental studies have confirmed this finding in the case of the small East African goats in Kenya and Red Maasai sheep [22,71]. However, Murray *et al*. [70] reported that Dwarf goats can be highly susceptible to experimental infection. West African Dwarf goats have been shown to be less trypanotolerant than Djallonke sheep due to a crucial introgression of genes of trypanosusceptible breeds into West African Dwarf goat populations, which possibly explains the loss of trypanotolerance in these goats. However, there are still sheep that are susceptible to trypanosomiasis.

## What is the Importance of Breeding for Trypanotolerance/Trypanoresistance in Low-input/Low-output Sheep Production Systems?

Smallholder sheep production has a crucial role in the livelihood of farmers and economies of developing countries, as they are among the primary producers of food (World Resources Institute) [71]. Other important roles sheep play in the livelihood of farmers are production of meat for consumption, manure, income, and wool, in addition to cultural and religious rituals. These sheep have highly diverse genetic resources that have low production performance levels [72]. However, commercial farmers attain high production performance because exotic breeds are selected for improved reproduction, growth, wool, and meat traits [73]. On the other hand, in smallholder sheep production systems, there is limited or no selection for traits such as disease resistance [74]. Studies of disease resistance in sheep include studies of resistance to ticks [75], pneumonia, foot rot, and nematodes [76]; few studies have reported on trypanotolerance [55,68,76].

Trypanosomiasis directly impacts livestock management, especially the numbers of livestock kept by farmers. Unfortunately, important population data, such as breed data, are difficult to obtain at both the national and the continental levels [76]. Geerts and Holmes [77] reported that each year an estimated more than 35 million doses of trypanocidal drugs are administered in Africa at an average purchase price of $1 per dose, which translates to $35 million per year spent in response to trypanosomiasis. However, the use of trypanocidal drugs to control and treat trypanosomiasis has negative consequences, including the scarcity of available drugs and the development of trypanosome drug resistance [77,78]. Furthermore, there is a lack of veterinary services in low-input/low-output sheep production systems, which results in a high degree of misuse of these drugs, leading to resistant strains of trypanosomes. Misuse results from a lack of experience in drug administration (incorrect routes, wrong drug choice, inconsistent administration intervals for prophylaxis, and disregarding manufacturers’ recommendations) [79]. In addition, the lack of veterinary services in these areas results in farmers acquiring drugs from unqualified service providers [80]. Breeding for trypanosome tolerance/resistance will help mitigate these limitations and help smallholder farmers maximize their productivity.

## National Small Stock Improvement Schemes (NSIS) and Community-based Breeding Programs (CBBPs) in Low-input/low-output Smallholder Systems

To breed for trypanosome tolerance/resistance in low-input/low-output sheep production systems, accurate records are needed, especially records of phenotypes to include in genomic selection and selection indices. Chagunda *et al*. [81] reported that farmers failed to keep records because of a lack of knowledge and education as well as being committed to other activities. It would be of great importance to empower such farmers with relevant knowledge that could help them improve their record keeping, which will ultimately improve their farming. Farmers can utilize technology in the form of smartphones to capture records [82], which has proved to be effective when used to share knowledge in South Africa [83].

This leads to another challenge that needs to be addressed, that is, the absence of a NSIS, which makes it almost impossible for sheep farmers to take part in genetic improvement programs [84]. An NSIS is a government initiative that aims at genetically improving economic production traits in a comprehensive approach while maintaining breed standards. The scheme serves as a basis for accurate recording of economically important traits in various sheep and goat breeds. Breeders then change their focus and prioritize traits on monetary value under natural production environments.

In addition, CBBPs are absent in most African countries. CBBPs for small stock are a possible option available to achieve genetic improvement in low-input/low-output small stock production systems. These programs aim at reducing the effect of inbreeding by controlling the rotation of rams, mating, and increasing flock size by bringing households together. Their success is based on the consideration of the views, needs, decisions, and active participation of sheep farmers from establishment to execution [85]. As reported by Wurzinger *et al*. [86], more than any form of financial support, satisfying the interests of smallholder farmers is crucial for the sustainability of genetic improvement programs. Smallholder farmers rear animals that possess disease-resistance traits [87,88]; however, few studies have been performed to unveil the richness of their genetic resources. This information can help in devising strategies for genetic improvement of sheep and goats. Animal breeding is used to improve populations of livestock by selecting the best animals of the current generation and using them as parents of the next generation. In recent years, small ruminant breeding strategies have largely focused on importing exotic breeds for crossbreeding [89]. The imported breeds include sheep breeds such as Merino, Hampshire, Bleu du Maine, Romney, Corriedale, Rambouillet Dorper, and Awassi; imported goat breeds include Toggenburg Anglo-Nubian, Saanen, and Boar. However, exotic breeds often underperform under the harsh conditions they find themselves in due to the effects and magnitude of genotype-by-environment interactions and extensive production system with limited supplementation of feed [87,90]. Furthermore, these genetic improvement programs have had no significant effects on goat and sheep productivity or on farmers’ and pastoralists’ livelihoods and the national economy at large [91]. However, farmer co-operatives or CBBPs where rams are shared can result in genetic improvement in smallholder farming systems, especially in the first-generation progeny (Table-1) [92-99]. These CBBPs have shown exceptional genetic progress in targeted production traits [85]. They have served as a working model for Animal Genetic Resource Management in developing countries such as Liberia to increase productivity and improve the livelihood of livestock keepers [92]. However, these programs have failed in other countries because of difficulties such as lack of participation of farmers in defining the breeding objective [86]. In South Africa, it has been reported that farmers in co-operatives do not receive support because the agricultural departments are not aware of their existence [100]. This shows the need for different stakeholders (farmers, researchers, educational institutions, private and public companies, as well as government departments) to come together to bring change in the smallholder farming system.

**Table 1:** Community-based breeding programs in Africa.

Country	Location/County	Reference
Ethiopia	Afar, Bonga, Horro and Menz	[93-95]
Liberia	Bong, Nimba and Grand Bassa	[92]
Kenya	Turkana, Marsabit and Isiolo	[96]
Tanzania	Kilimanjaro	[97]
Malawi	Zombwe, Magoti, Mkwinda, Mzimba and Nsanje	[98,99]
Uganda	Buseruka and Namalu	[98]

## Genomics Selection for Trypanosomiasis Resistance

Artificial selection and natural selection are expected to impose pressure on specific regions of the genome [101]. The genomic regions with elevated homozygosity in the population under selection are called selection signatures or selective sweep [102,103]. Detection of selection signatures in a particular sheep population can assist in identifying genes and beneficial mutations that carry a selective advantage. Knowledge of selection signatures and genetic diversity is very crucial for conservation programs and breed improvement [104]. Due to advances in high-throughput technologies, genome-wide detection of selection signatures has become widespread in recent years, with studies in sheep increasing because of their economic importance [105-107]. These studies help to facilitate the identification of candidate genes under selection that is associated with economically important traits in sheep populations.

Single-nucleotide polymorphism (SNP) genotyping is one of the most common methods of studying selection signatures, as SNPs are abundant in the genome, genetically stable, and convenient for high-throughput automated analysis. The application of SNP-based parentage becomes cost-effective if it forms a part of routine genotyping. In developing countries, where routine genotyping for genomic selection has not yet been made a standard practice, microsatellite markers are still employed for verification of parentage. The OvineSNP50 Bead Chip (Illumina Inc., San Diego, CA) is the most comprehensive SNP genotyping array [108]. It has been validated in 75 sheep breeds that are economically important worldwide [109]. Several statistical methods have been developed specifically for detecting selection signatures, using SNP data based on site linkage disequilibrium, frequency spectrum, population differentiation, and reduced local variability [110]. Linkage disequilibrium-based methods, such as integrated haplotype score, target homozygous regions with high frequency haplotypes generated by selective sweeps. When two haplotypes share a recent common ancestor, contiguous lengths of homozygous genotypes emerge within an individual and are known as runs of homozygosity (ROHs) [111]. In addition to being used to calculate levels of genomic inbreeding in animals, ROHs are used to identify the selective regions that have decreased variation relative to the genome average [112,113]. Using genome-wide SNP data, signatures of positive selection for trypanotolerance have been identified [107].

Whole-genome sequencing (WGS) provides the most comprehensive collection of genetic variation of individuals [114]. It can be used to understand population diversity and to identify genes related to traits of interest. Yang *et al*. [115] used WGS technology to identify genes related to adaptation traits in sheep adapted to various environments. In addition, Yaro *et al*. [76] revealed regions of heterozygosity that correspond to alleles shared between *Trypanosoma brucei rhodesiense* and *Trypanosoma brucei gambiense*. In addition, WGS identified more than a million novel genomic variants of the Sahelian and Djallonke sheep breeds, which suggests that both sheep breeds represent unique genetic resources and are important for world sheep diversity [76]. WGS may become the most effective genotyping method if the sequencing costs continue to decrease, despite the challenges to the current efficient use of WGS data [116].

Applications of genomic technology for small stocks also use copy number variation (CNV) data. Detection and analysis of CNV by WGS are feasible methods because of the lower cost of next-generation sequencing techniques compared with other methods. Analysis of CNV on genome-wide SNP data can lead to the identification of chromosomal regions containing structural variations affecting complex traits [117], such as trypanosomiasis resistance traits. CNV is a crucial source of genetic variation in an individual, as it covers more genomic regions than single SNPs. It alters gene expression and protein function and changes the phenotype of an individual due to duplication, translocation, inversion, and deletion of genes in the CNV regions [118]. However, the current focus is on the identification of CNVs associated with complex traits [119,120].

Comparative analysis of genomic information is also a powerful tool for understanding the genomic drivers of trypanosome susceptibility. Trypanosome challenge has shaped the formation of native African domestic ruminant populations by applying environmental and selective pressure to the genome of small African ruminants [7,121]. Recently, QTLs associated with trypanotolerance traits in Djallonke sheep were reported [107]. The authors compared the orthologous genomic regions in Djallonke sheep with the genomic regions associated with QTLs for trypanotolerance in West African N’Dama cattle reported by Hanotte *et al*. [45]. Their findings revealed only one orthologous candidate gene, located on chromosome 4 (BTA4) (CAV1). These results are promising and demonstrate the ability of comparative analysis of WGS information to yield candidate loci of functional relevance. Further analysis is required to elucidate the significance of this finding and validate the functional relevance of the candidate gene in both species. The advantage of genomics to smallholder farmers may be the characterization of their animals, and this benefit may hold great potential in terms of gene introgression into exotic breeds. For example, hypocretin receptors in trypanotolerance, the Bovine Leukocyte Antigen complex in tick resistance, is the unique haplotype identified in indigenous breeds [87] and could benefit commercial farmers if introduced into their livestock.

## Challenges of Using Genome-wide Association Studies (GWAS) to Identify Loci underlying Variation in Trypanosomiasis Resistance in Low-input/low-output Smallholder Systems

GWAS identify associations between phenotype and genotype by testing genetic variants across the genomes of many individuals [122]. They have transformed the field of complex disease genetics over the past decade by providing numerous compelling associations for animal and human complex traits as well as diseases of animals and humans [123]. GWAS are advantageous in that they can overcome the approach that utilizes candidate genes through which significant results are sometimes not obtained due to the incomplete or wrong choice of candidate genes. The first GWAS that became a success was published in 2005. It investigated genome-wide scans of polymorphisms associated with age-related macular degeneration in humans and found two SNPs with remarkably altered allele frequencies compared to controls [124]. In livestock, GWAS have been shown to be an ideal method for identifying genes associated with different phenotypes and explaining the mechanisms of complex traits [125]. GWAS have been performed in various sheep strains worldwide (Table-2) [76,126-133].

**Table 2:** Genome-wide association studies applied in different sheep breeds to identify genes associated with traits of economic importance.

Study title	Sheep breed	Country/Continent	Reference
Analysis of pooled genome sequences from Djallonke and Sahelian sheep of Ghana reveals co-localization of regions of reduced heterozygosity with candidate genes for disease resistance and adaptation to a tropical environment	Djallonke and Sahelian sheep	Ghana	[76]
Selection Signatures in Worldwide Sheep Populations	African Dorper, African White Dorper, Afshari Altamurana, Australian Coopworth, Australian, Industry Merino, Australian Merino, Australian Poll Dorset, Australian Poll Merino, Australian Suffolk Bangladeshi BGE, Bangladeshi Garole, Barbados Black Belly, Black-Headed Mutton, Border Leicester Boreray, Brazilian Creole, Bundner Oberlander Sheep, Castellana, Changthangi, Chinese Merino Chios, Churra, Comisana, Cyprus Fat Tail, Deccani Dorset Horn, East-Friesian Brown, East-Friesian White, Engadine Red Sheep, Ethiopian Menz Finn sheep, Galway, Garut, German Texel, Gulf Coast Native, Indian Garole, Irish Suffolk, Karakas, Leccese, MacArthur Merino, Meat Lacaune, Merino Landschaf, Milk Lacaune, Moghani, Morada Nova, Namaqua Afrikaner, New Zealand Romney, New, Zealand Texel, Norduz, Ojalada, Old Norwegian Spaelsau, Qezel, Rambouillet, Rasa Aragonesa, Red Maasai, Ronderib Afrikaner, Sakiz, Santa Ines, Sardinian Ancestral Black, Scottish Blackface, Scottish Texel, Soay, Spael-coloured Spael-white, St. Elizabeth, Sumatra, Swiss Black-Brown Mountain Sheep, Swiss Mirror Sheep, Swiss White Alpine Sheep, Tibetan, Valais Blacknose Sheep, Valais Red Sheep, Wiltshire	Africa, Asia, Europe, Australia, New Zealand, Europe, North America	[126]
A Genome Wide Survey of SNP Variation Reveals the Genetic Structure of Sheep Breeds	Dorper, Suffolk, Blackface, Charollais, German Mountain Brown, Javanese Thin Tail, Italian Sarda, Merino, Poll_Dorset, Rambouillet, Red Masai, Romney, Soay, Sumatran Thin Tail, Texel, Tibetan, Finsheep, Katahdin, Romanov, Namaqua Afrikaner, Ronderib Afrikaner, Bighorn, Thinhorn	Africa, Asia, Europe, Australia, New Zealand, Europe, North America	[127]
Candidate genes for productivity identified by genome-wide association study with indicators of class in the Russian meat merino sheep breed, Vavilov Journal of Genetics and Breeding	Merino sheep	Russia	[128]
Whole-genome resequencing of wild and domestic sheep identifies genes associated with morphological and agronomic traits, Nature Communications	Domestic sheep (*O. aries*) and wild sheep (*Asiatic mouflon*, *Ovis orientalis*)	Asia, Europe, Africa, and the Middle East	[129]
Genome-Wide Association Study Identifies New Candidate Markers for Somatic Cells Score in a Local Dairy Sheep	Valle del Belice breed	Sicily	[130]
Genome-wide association mapping identifies the genetic basis of discrete and quantitative variation in sexual weaponry in a wild sheep population	Wild Soay sheep (*O. aries*)	Scotland	[131]
A genome-wide association study reveals candidate genes for the supernumerary nipple phenotype in sheep (*O. aries*)	Wadi sheep	China	[132]
Preliminary genome-wide association study for wet-dry phenotype in smallholder ovine populations in South Africa	Dorpers (Dorpersm) and White Dorpers	South Africa	[133]

SNP=Single-nucleotide polymorphism, *O. aries*=*Ovis aries*

Several challenges that make undertaking GWAS in low-input smallholder systems difficult. First, the majority of smallholder farmers do not keep records of traits of their flocks, which makes obtaining accurate indicator traits difficult [134,135]. In addition, it is difficult to describe breed characteristics because of the extensive production system under which communal farmers raise their animals, which is characterized by a lack of recorded pedigree and production records [134,136]. At present, the available knowledge of trypanotolerance is not sufficient for developing crossbreeding programs or for efficient selection of the right traits. Therefore, the spread of beneficial effects by selection or crossbreeding will be based on the ability to precisely characterize the trypanotolerant phenotype at the genetic level. Without baseline information, such as flock records, it is challenging to conduct GWAS studies to identify chromosomal regions that harbor genes that contribute to trypanotolerance.

Another challenge is the need for large sample sizes to provide sufficiently powerful analyses to generate robust and reproducible results [135]. Researchers have opted for selective DNA pooling or selective genotyping to reduce the number of individuals to be genotyped; however, it has been shown that these methods can lead to loss of information on specific individuals in the study [137]. Financial constraint is one of the hurdles that hinder conducting GWAS in communal areas and limit the ability of bioinformatics to deal with big data. For example, a study by Noyes *et al*. [19] identified two genes that enable African N’Dama cattle to fight against AAT. When analyzing the big datasets generated from N’Dama cattle, the authors were faced with computational challenges that led them to outsource the services of the University of Manchester School of Computer Science [138].

GWAS can be based on SNP chip data. In a study by Smetko *et al*. [56], trypanotolerance candidate regions were discovered using a selection signature method based on differences in allele frequencies between trypanotolerant African taurine and other world breeds. The authors targeted a small number of SNP genotypes in the candidate regions. However, they mentioned that part of their data was being analyzed using a commercial chip from Illumina, which covers a wide range of SNPs (approximately 800,000), since the cost of high-density SNP chip genotyping is starting to decrease. However, the cost of generating large enough numbers of dense genotypic profiles is still high in developing countries, making it difficult to evaluate local breeds. Hence, alternative methods, such as imputation, are used to overcome this shortcoming. This approach uses higher-SNP chips to genotype only key individuals in a population to form the foundation from which animals that are genotyped with low-density SNP are imputed to the same density as the former [139]. For this method, SNP markers that are not genotypes are generated *in silico*, providing crucial genotypic information based on predictive model-based algorithms.

Moreover, data sets from different studies can be subjected to rigorous data checks and put together, and data imputation can be used as a tool to avoid false-negative and biased results, since SNPS have been shown to be biallelic in nature [140]. This method was not possible with microsatellite technology because it resulted in high error rates. Therefore, many researchers prefer using SNPs as genetic markers [141]. However, it has been reported that the accuracy of genotype imputation is affected by various factors, such as the genetic relationship between the validation populations and the reference [142], the size of the reference population [142], imputation algorithms, the SNP density of the target panel [143], and the sequencing depth [144]. Ventura *et al*. [143] found that it was possible to improve the accuracy of imputation by two-step imputation using a multibreed sheep population genotyped with three SNP panels: 5 K, 50 K, and 600 K. In a study by Ye *et al*. [145], increasing the sequencing cost resulted in higher imputation accuracy. The authors explained that at a fixed sequencing cost, the optimal imputation strategy should consider the imputation algorithms, the sequencing depth and size of the reference population, and the density of the marker, as well as the population structure of the target population. At present, no studies have utilized imputation to investigate rare alleles in trypanosomiasis resistance in sheep in low-input/low-output smallholder systems, especially for regions under selection pressure.

## Conclusion

Several control methods are used against trypanosomiasis, including trypanocidal drugs and insecticides. However, these methods are not without faults, which calls for alternative methods to be employed. Breeding for disease resistance can be used as an alternative method in combination with other integrated control strategies, such as reducing the proximity of livestock to reservoir hosts to control trypanosomiasis. It is necessary to consider the use of trypanotolerant breeds of small stock as a sustainable approach to livestock development in areas challenged by the tsetse fly. In addition, the identification of SNPs associated with transcriptome challenges could yield positive results for developing customized chips for low-input/low-output farming systems. Ultimately, genomic tools can be used as an alternative means to control trypanosomiasis. There is a high possibility that this control strategy can be implemented in the majority of African countries to breed for trypanosome tolerance/resistance in low-input/low-output sheep production systems. However, it will be crucial to start by collecting baseline information, such as pedigree and production records, before commencing breeding. Routine SNP genotyping of livestock populations in these production systems would also assist with genetic data important for breeding. Finally, improving productivity in low-output sheep production systems could be a pathway out of poverty for smallholders in Africa.

## Authors’ Contributions

DPM: Conceived, planned, and executed the writing of the manuscript.
